# 

**Published:** 1950-10

**Authors:** 


### Local Medical Notes

### Appointments

Mr. H. J. Croot, Professor of Surgery, Makerere College, East Africa.

*Bath:*

Blair, B. E., M.B., F.R.C.S., M.R.C.O.G.—Consultant Obstetrician and Gynaecologist.

Pugh, D. W., D.S.C., M.D., M.R.C.P.—Consultant Physician.

Sames, C. P., M.S., F.R.C.S.—Consultant Surgeon.

*Bristol:*

Airth, G. R., M.B., D.M.R.D.—Consultant Radiologist.

Early, D. F. M., D.P.H., D.P.M.; Leitch, A., M.D., D.P.M.—Consultant Psychiatrists.

Roberts, A. T. M., M.D., M.R.C.P.—Consultant Chest Physician.

Maggs, R., M.B., D.P.M.—Physician in Psychiatry, Bristol Mental Hospital.

Heaton-Ward, W. A., M.B., D.P.M.—Deputy Medical Superintendent, Hortham and Brentry Colony.

*Exeter:*

Caldwell, K. P. S., M.B., F.R.C.S.—Consultant Surgeon.

Powell, K. J., M.B., D.A.—Consultant Anaesthetist.

*North Gloucestershire:*

Little, B. R., M.R.C.S., D.A.—Consultant Anaesthetist.

Dr. R. E. Bowers.—Consultant Dermatologist.

Mr. S. A. Bond.—Consultant Gynaecologist.

*Plymouth:*

Blair, R. A., M.D., D.P.M.—Consultant Psychiatrist.

Sweet, R. D., M.B., M.R.C.P.—Consultant Dermatologist.

*West Cornwall:*

Grant, R. N., F.R.C.S.—Consultant Orthopaedic Surgeon.

Lewis, J. K., M.B., D.A.—Consultant Anaesthetist.

St. John-Brooks, W. H. S., M.B., M.R.C.P.—Consultant Physician.

Simons, P. N., M.B., F.R.C.S., M.R.C.O.G.—Consultant Obstetrician and Gynaecologist.

### Degrees and Diplomas

**Royal Colleges of Physicians and Surgeons (Conjoint Board).**—M.R.C.S., L.R.C.P.: R. J. Ancill, W. A. W. Dutton, R. F. Harvey, R. Hill, D. T. I. Stuttaford.

